# Comparative Genomic Hybridization to Microarrays in Fetuses with High-Risk Prenatal Indications: Polish Experience with 7400 Pregnancies

**DOI:** 10.3390/genes13040690

**Published:** 2022-04-14

**Authors:** Katarzyna Kowalczyk, Magdalena Bartnik-Głaska, Marta Smyk, Izabela Plaskota, Joanna Bernaciak, Marta Kędzior, Barbara Wiśniowiecka-Kowalnik, Marta Deperas, Justyna Domaradzka, Alicja Łuszczek, Daria Dutkiewicz, Agata Kozar, Dominika Grad, Magdalena Niemiec, Kamila Ziemkiewicz, Róża Magdziak, Natalia Braun-Walicka, Artur Barczyk, Maciej Geremek, Jennifer Castañeda, Anna Kutkowska-Kaźmierczak, Paweł Własienko, Krystyna Jakubów-Durska, Marzena Dębska, Anna Kucińska-Chahwan, Szymon Kozłowski, Boyana Mikulska, Tadeusz Issat, Tomasz Roszkowski, Agnieszka Nawara-Baran, Agata Runge, Anna Jakubiuk-Tomaszuk, Anna Kruczek, Ewa Kostyk, Grzegorz Pietras, Janusz Limon, Jerzy Zwoliński, Karolina Ochman, Tomasz Szajner, Piotr Węgrzyn, Mirosław Wielgoś, Maria Sąsiadek, Ewa Obersztyn, Beata Anna Nowakowska

**Affiliations:** 1 Department of Medical Genetics, Institute of Mother and Child, Kasprzaka 17a, 01-211 Warsaw, Poland; magdalena.bartnik@imid.med.pl (M.B.-G.); marta.smyk@imid.med.pl (M.S.); izabela.plaskota@imid.med.pl (I.P.); joanna.bernaciak@imid.med.pl (J.B.); marta.kedzior@imid.med.pl (M.K.); barbara.wisniowiecka@imid.med.pl (B.W.-K.); marta.deperas@imid.med.pl (M.D.); justyna.domaradzka@imid.med.pl (J.D.); alicja.luszczek@imid.med.pl (A.Ł.); daria.dutkiewicz@imid.med.pl (D.D.); agata.kozar@imid.med.pl (A.K.); dominika.grad@imid.med.pl (D.G.); magdalena.niemiec@imid.med.pl (M.N.); kamila.ziemkiewicz@imid.med.pl (K.Z.); roza.magdziak@imid.med.pl (R.M.); natalia.braun-walicka@imid.med.pl (N.B.-W.); artur.barczyk@imid.med.pl (A.B.); maciej.geremek@imid.med.pl (M.G.); jennifer.castaneda@imid.med.pl (J.C.); anna.kutkowska@imid.med.pl (A.K.-K.); pawel.wlasienko@imid.med.pl (P.W.); krystyna.jakubow@imid.med.pl (K.J.-D.); ewa.obersztyn@imid.med.pl (E.O.); beata.nowakowska@imid.med.pl (B.A.N.); 2 1st Department of Obstetrics and Gynecology, Medical University of Warsaw, pl. S. Starynkiewicza 1/3, 02-015 Warsaw, Poland; marzena@debska.me (M.D.); mwielgos@wum.edu.pl (M.W.); 3 Department of Gynecology Oncology and Obstetrics, Medical Centre of Postgraduate Education (CMKP), Czerniakowska 231, 00-416 Warsaw, Poland; ankakucinska@wp.pl (A.K.-C.); tomcior1@gmail.com (T.R.); 4 Clinic of Obstetrics and Gynaecology, Institute of Mother and Child, Kasprzaka 17a, 01-211 Warsaw, Poland; szymon.kozlowski@imid.med.pl (S.K.); boyana.mikulska@imid.med.pl (B.M.); tadeusz.issat@imid.med.pl (T.I.); 5 Specialist Gabinets Volumed, Zdrowa 1/3, 31-216 Krakow, Poland; anawarabaran@gmail.com; 6 Genetic Department, Ludwik Rydygier Collegium Medicum in Bydgoszcz, Nicolaus Copernicus University of Torun, 85-067 Bydgoszcz, Poland; agata.run@wp.pl; 7 Medical Genetics Unit, Mastermed Medical Center, 15-302 Bialystok, Poland; ajaktom@gmail.com; 8 Genetic Counselling Unit Kostyk and Kruczek, Henryka Wieniawskiego 64, 31-436 Krakow, Poland; kruczekanna@poczta.onet.pl (A.K.); ewa@kostyk.pl (E.K.); 9 Department of Obstetrics and Perinatology, Medical University of Lublin, Jaczewskiego 8, 20-090 Lublin, Poland; piegrze@gmail.com; 10 Prenatal Unit, Medical University of Gdansk, Marii Sklodowskiej-Curie 3a, 80-210 Gdansk, Poland; jlimon@gumed.edu.pl; 11 Department of Obstetrics, Hospital of St. Families in Warsaw, Madalinskiego 25, 02-544 Warsaw, Poland; jerzy.zwolinski.jzs@gmail.com; 12 Clinics and Medical Laboratories INVICTA, Genetics Clinic, Rajska 10, 80-850 Gdansk, Poland; karolina.ochman@invicta.pl; 13 Specialist Hospital Pro-Familia, Witolda 6B, 35-302 Rzeszow, Poland; tomisek@interia.pl; 14 Department of Obstetrics, Perinatology and Gynaecology, Faculty of Health Sciences, Medical University of Warsaw, Zwirki i Wigury 63a, 02-091 Warsaw, Poland; piotr.wegrzyn@wum.edu.pl; 15 Department of Genetics, Wroclaw Medical University, K. Marcinkowskiego 1, 50-368 Wroclaw, Poland; maria.sasiadek@umw.edu.pl

**Keywords:** microarray, prenatal diagnosis, CMA, aCGH

## Abstract

The aim of this study was to determine the suitability of the comparative genomic hybridization to microarray (aCGH) technique for prenatal diagnosis, but also to assess the frequency of chromosomal aberrations that may lead to fetal malformations but are not included in the diagnostic report. We present the results of the aCGH in a cohort of 7400 prenatal cases, indicated for invasive testing due to ultrasound abnormalities, high-risk for serum screening, thickened nuchal translucency, family history of genetic abnormalities or congenital abnormalities, and advanced maternal age (AMA). The overall chromosomal aberration detection rate was 27.2% (2010/7400), including 71.2% (1431/2010) of numerical aberrations and 28.8% (579/2010) of structural aberrations. Additionally, the detection rate of clinically significant copy number variants (CNVs) was 6.8% (505/7400) and 0.7% (57/7400) for variants of unknown clinical significance. The detection rate of clinically significant submicroscopic CNVs was 7.9% (334/4204) for fetuses with structural anomalies, 5.4% (18/336) in AMA, 3.1% (22/713) in the group of abnormal serum screening and 6.1% (131/2147) in other indications. Using the aCGH method, it was possible to assess the frequency of pathogenic chromosomal aberrations, of likely pathogenic and of uncertain clinical significance, in the groups of cases with different indications for an invasive test.

## 1. Introduction

Prenatal diagnosis is one of the most important achievements of modern perinatology. Intrauterine imaging of the fetus enables the early detection of a significant number of malformations and creates a chance for intrauterine treatment or surgical intervention immediately after birth. Therefore, according to the recommendations of the Polish Society of Gynecologists and Obstetricians regarding the management of prenatal diagnosis, all pregnant women should be offered prenatal screening tests that determine the risk of chromosomal abnormalities in the fetus. Invasive genetic tests should be performed when screening tests show a higher risk of having a child with a genetic defect than the normal population.

The main indications for an invasive prenatal diagnosis have mainly included ultrasound anomalies, high-risk for serum screening, family history of genetic disorders or birth defects, and advanced maternal age (AMA) (over 35 years old). Currently, the first-choice method in prenatal diagnosis is the method of comparative genomic hybridization to microarrays (aCGH). The variability of the etiology and clinical heterogeneity of these disorders require high-sensitivity and the whole genome method.

Similar indications can be found in the available literature. Luo X. et al., used chromosome microarray analysis (CMA) to evaluate 1256 prenatal cases (between 15–33 weeks of gestation) with clinical indications: non-structural ultrasound anomalies (*n* = 132), structural ultrasound anomalies (*n* = 283), high-risk for maternal serum markers (*n* = 44), abnormal non-invasive prenatal test results (*n* = 143), family history of genetic disorders (*n* = 288), AMA (*n* = 328), and other indications (*n* = 38) [1]. In another group led by Xiang J. et al., the research was carried out on a group of 5000 pregnancies with the following indications: anomaly on ultrasonography (*n* = 1055), advanced maternal age (*n* = 1784), abnormal result on maternal serum screening (*n* = 1199), abnormal NIPT results (*n* = 515) and other indications (*n* = 709). The mean age of the pregnant women qualified for the study was 32.04 years (range: 18–49 years), and the mean gestational age was 21.28 weeks (range: 9–34 weeks) [2].

The introduction of the CMA method in the late 90s, revolutionized cytogenetic diagnostics, enabling detailed results to be obtained in a short time. As a result, in recent years it has been widely used in prenatal diagnostics [3,4,5].

Congenital malformations of the fetus concern ~70% of spontaneous miscarriages, ~2–3% of live births and ~20% of stillborn. They are also one of the leading causes of infant mortality. Currently, it is estimated that about 35% of congenital malformations are genetically determined. Among the genetic causes of congenital malformations, chromosome aberrations account for approximately 24% of cases in live births and approximately 50% of spontaneous abortions [3].

Congenital malformations divide into isolated malformations or multiple malformations, which are accounted for 2/3 and 1/3 of all malformations, respectively.

It is estimated that about 6–7% of congenital malformations are caused by submicroscopic genome imbalance, which can only be detected using the microarray technique. Additionally, in ~1% of fetuses with a congenital malformation, alterations in the number of copies of DNA sequences of unknown clinical significance are found [2].

In the available literature, the detection rate of pathogenic imbalances between the different clinical groups is variable. For example, Luo X. et al., showed overall prenatal diagnostic yield was (7.8%) of 1256 pregnancies. Clinically significant genomic aberrations were identified in 1.5% of analyzed patients with non-structural ultrasound anomalies, 12.7% in fetus with structural ultrasound anomalies, 4.5% at high-risk for biochemic test, 26.6% at high-risk for NIPT, 3.8% with a burdened family history, and 2.1% with AMA [2].

The use of the aCGH microarray method to diagnose fetuses with abnormal ultrasound results is recommended by European scientists. According to the American Congress of Obstetricians and Gynecologists (ACOG) and the Society for Maternal-Fetal Medicine (SMFM), CMA should be used as a first-tier diagnostic test for all fetuses, both with the presence of fetal structural abnormalities and in patients who wish to continue prenatal diagnosis in the case of normal ultrasound of the fetus. Additionally, this test can be considered in all women undergoing prenatal screening, regardless of their age [6,7]. While the recommendations of the Canadian College of Medical Geneticists (CCMG) recommend the use of CMA when multiple fetal malformations or nuchal translucency (NT) ≥3.5 mm is detected after normal rapid screening for the most common aneuploidies [8].

Based on the above recommendations, copy number variations detected in the microarray CGH study, can be classified as pathogenic, likely pathogenic, likely benign, benign and of unknown significance (VOUS). To establish the origin of aberrations, a routine karyotype analysis by the GTG technique (G-bands after trypsin and Giemsa), FISH (Fluorescence In Situ Hybridization), aCGH, or MLPA method (Multiplex Ligation-dependent Probe Amplification) should be performed on the fetus’s parental material, depending on the type and size of the variant, and the methods available in the laboratory. Interpretation of the clinical significance of unknown CNVs is often very complicated and there is no single general rule or algorithm for the interpretation of test results.

The greatest diagnostic challenge in prenatal testing are the aberrations of unknown clinical significance. Therefore, following the recommendations issued by the European Cytogenetics Association, these abnormalities should not be reported in prenatal results [9]. As opposed to European recommendations, the CCMG indicates that variants of unknown importance, greater than a 500 kb in the case of deletions and 1 MB for duplications, should be shown in the prenatal results [8].

The VOUS, unlike likely pathogenic aberrations, do not involve genes of known pathogenicity and are absent in a few cases in the general population as likely benign. The numbers of VOUS will decrease as their importance is published in the medical literature and publicly available databases. It is assumed that the VOUS frequency remains quite variable. For example: Song T. et al., reported variants of unknown importance (VOUS) in 14/190 (7.37%) cases, Lee M.Y. et al., in 4/32 (12.5%), Yan Y. et al., in 4/76 (5.3%), and Wu X.L. et al., in 2.9% (3/104) of cases [10,11,12,13]. This difference can be explained by considering the different resolution of the microarray platform used by the authors and the differences in the interpretation of the meaning of the same VOUS [14]. In addition, a variant of unknown significance may in fact be pathogenic but has been classified as a VOUS due to insufficient data to determine its actual pathogenic significance. Therefore, a better understanding of VOUS are necessary to ensure appropriate prenatal genetic counseling [10].

To date, many studies evaluating the performance of CMA in prenatal diagnosis have been published. A study by the National Institute of Child Health and Human Development (NICHD) showed clinically significant CNV in 6% of fetuses with normal karyotype and anatomical defects [15]. In the study by Srebniak et al., a group of 1033 fetuses with abnormalities found in ultrasound was analyzed and pathogenic CNV found in 5.5% of cases [16]. In a larger study of 5000 fetuses, the prevalence of CNV was found to be 6.6% in 2462 cases with ultrasound abnormalities [17].

We present the results of a chromosomal microarray analysis in a larger cohort of 7400 cases diagnosed in the single center, the Institute of Mother and Child, from January 2014 to September 2021. We identified pathogenic aberrations and likely pathogenic abnormalities in 505 fetuses, 57 CNVs of unknown clinical significance and 17 CNVs that were classified as likely benign. Indications for the study included the following groups: prenatally diagnosed ultrasound abnormalities, high risk for screening, thickened nuchal translucency, family history of genetic abnormalities or congenital abnormalities, and advanced maternal age (AMA).

## 2. Materials and Methods

**Figure 1:**
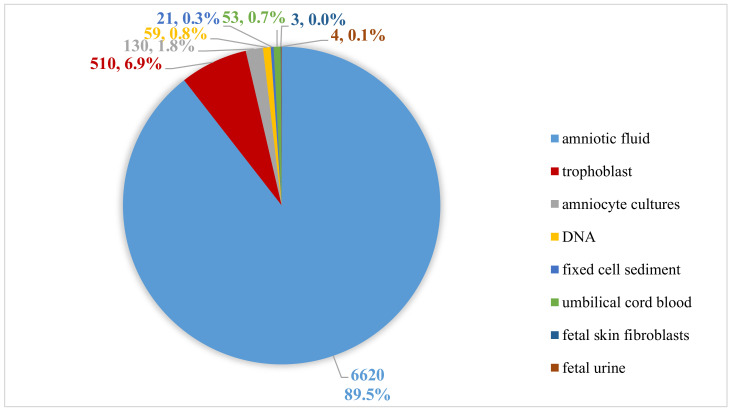
Types of materials used for extraction DNA.

In the aCGH study, the DNA was isolated from amniotic fluid (*n* = 6620), trophoblast (*n* = 510), amniocyte cultures (*n* = 130), fixed cell sediment (*n* = 21), umbilical cord blood (*n* = 53), fetal skin fibroblast (*n* = 3) or fetal urine (*n* = 4) using the Sherlock kit (A&A Biotechnology) (Figure 1). The tests were performed on patients between 11 and 35 weeks of pregnancy. The amniocentesis was performed between 13–23 weeks of pregnancy, trophoblast biopsy between the 11th and 13th, and cordocentesis between 23–35 weeks of pregnancy.

### 2.1. Sample Types and DNA Isolation

The research was approved by the Bioethics Committee of the Institute of Mother and Child in Warsaw. All pregnant women decided to undergo aCGH testing after receiving genetic counselling and signing informed consent prior to invasive prenatal testing. The study group included a total of 7400 samples (Figure 1). The mean age of the pregnant women was 35 years (range: 18–49 years). Amniotic fluid (*n* = 6545), trophoblast (*n* = 495), amniocyte cultures (*n* = 114), fixed cell sediment (*n* = 16), umbilical cord blood (*n* = 41), fetal skin fibroblast (*n* = 3) or fetal urine (*n* = 4) and sample DNA (*n* = 56) were collected and successfully analyzed at the Department of Medical Genetics of the Institute of Mother and Child in the period from January 2014 to September 2021. Due to the poor quality of the material provided to us for DNA isolation, too early amniocentesis, incorrectly performed chorionic villus sampling and failures in cell culture, in 126 cases we obtained very poor data, which did not allow us to issue an informative result. Table 1 shows the number of tests performed in a particular year along with the number of correct, incorrect, and non-informative results. In cases where it was required, parents blood samples were taken to determine the origin of the identified aberrations.

In all samples of amniotic fluid, trophoblast and fetal urine except umbilical cord blood, cell cultures were established for DNA preservation and conventional karyotype determination. Genomic DNA was immediately isolated from the fresh material. When amniotic fluid (AF) and chorionic villus sampling (CVS) was visibly contaminated, we used a cell culture that eliminates maternal cells and stimulates the growth of fetal cells and then DNA was isolated from cultured cells. The CVS villi were separated from maternal tissue under a microscope to minimize maternal cell contamination (MCC). Two to four villi were used for DNA extraction. Sample DNA was isolated from 2 to 5 mL of amniotic fluid. The AF samples were centrifuged. For CVS, incubation at 56 °C with 20 µL of proteinase K, water, and tissue lysis buffer (L1.4 buffer) was performed for at least 1 h for efficient digestion and lysis of the entire sample. For AF samples, incubation at 56 °C with 20 µL of proteinase K, water, and lysis buffer (L1.4 buffer) was performed for 45 min. Genomic DNA was extracted using a DNA isolation kit (Sherlock A&A Biotechnology, Poland) according to the manufacturer’s instructions.

### 2.2. Genomic Array Platform (Array Comparative Genomic Hybridization (Array CGH) Analysis and Interpretation)

The aCGH was performed using an 8x60K microarray from Oxford Gene Technology (CytoSure ISCA, v2 and v3, Oxford, UK). The array used in this study contains 60-mer oligonucleotide probes covering the entire genome with an average spatial resolution of 120 kb. The CGH methodology description is available in the manufacturer’s website. All genomic coordinates are based on a reference genome (NCBI37/hg19). The data analysis was performed using CytoSure Interpret Software (Oxford Gene Technology, Oxford, UK) and a circular binary segmentation algorithm. The calling thresholds were the deviation of the binary cyclic segmentation (CBS) segment from a zero-log ratio of +0.30 for duplication and −0.5 for deletion. Our results were classified using the CytoSure Interpret Software (Oxford Gene Technology, Oxford, UK). Quality control measures were monitored using CytoSure Interpret Software (Oxford Gene Technology). The microarray used in this analysis does not contain SNP probes, does not detect polyploidy, inversion, balanced translocation regions of absence of heterozygosity.

In this study, we report the detection rate of chromosomal microarray analysis in the detection of fetal chromosomal aberrations among 7400 fetuses with congenital malformations, high risk for screening, thickened nuchal translucency, family history of genetic abnormalities or congenital abnormalities, and advanced maternal age (AMA) in Poland and evaluate the additional diagnostic yields produced by CMA for different referral indications (Table 2).

### 2.3. CNV Classification

The clinical relevance of each copy number variant should be considered individually using the general CNV classification. In our study, as recommended, we used five categories to classify the detected aberrations: pathogenic, likely pathogenic, variants of unknown significance (VOUS), possibly benign and benign:

1. Pathogenic aberrations: CNV was classified as pathogenic if it was a large aberration of several MB, or was associated with known microdeletion/microduplication syndromes or contained known genes responsible for a specific pathology and had been previously described in specific clinical disorders.
2. Likely pathogenic aberrations: CNVs that have not yet been described or have been rarely described and contain some gene/genes whose function is known and most likely may be responsible for the clinical features of the patient.
3. Variants of unknown significance (VOUS): This category includes CNVs that did not have a clearly defined clinical significance at the time of publication of the study results. These changes were not included in the prenatal outcomes because the function of the genes in this region was unknown or the cause of the abnormal ultrasound examination was difficult to determine. In our study, parental inheritance of VOUS was not specified.
4. Likely benign aberrations: CNVs that occur in healthy people and have only been described in a few cases in the general population, but do not represent a common polymorphism. CNV interpreted as possibly mild was not reported in a result of the study.
5. Benign aberrations: do not affect the phenotype (polymorphisms occurring in the general population), which include: aberrations in the region of segmental duplication, aberrations that do not contain genes, aberrations in regions containing dose-insensitive genes frequently repeated in the Polish population and known aberrations as copy number variants described in the Database of Genomic Variants (http://dgv.tcag.ca/dgv/app/home (accessed on 3 January 2022)) (path: DGV Gold Standard Variants).

All detected copy number variants (CNVs) were systematically assessed for clinical significance against those in the scientific literature and available databases: OMIM (http://www.ncbi.nlm.nih.gov/omim, (accessed on 3 January 2022)), Clinical Structural Variants (https://www.ncbi.nlm.nih.gov/dbvar/, (accessed on 3 January 2022)), Database of Genomic Variants (http://projects.tcag.ca/variation/ (accessed on 3 January 2022)), Ensembl (https://www.ensembl.org/index.html, (accessed on 3 January 2022)), and DECIPHER (http://decipher.sanger.ac.uk/, (accessed on 3 January 2022)).

## 3. Results

A total of 7400 samples were analyzed by aCGH from January 2014 to September 2021, of which 2010 samples yielded abnormal results (2010/7400, 27.2%), including 1431 cases with numerical chromosome anomalies (1431/7400, 19.3%) and 579 cases with CNV (579/7400, 7.8%) including pathogenic, likely pathogenic, VOUS and likely benign CNVs (Table 2). Benign, likely benign, and VOUS CNVs, were not reported in our results.

Among 1160 cases with numerical chromosome anomalies, trisomy 21 (578/7400, 7.8%) was the most common type, trisomy 18 (295/7400, 4%), trisomy 13 (130/7400, 1.7%), monosomy chromosome X (119/7400, 1.6%) and polyploidy (38/7400, 0.5%). A total of 135 CNVs (135/579, 23.3%) were inherited from a parent (97 maternal and 38 paternal) and 119 CNVs (119/579, 20.6%) were de novo in origin. A total of 446 CNVs (446/579, 77%) were classified as pathogenic (P), 59 as likely pathogenic (LP) (59/579, 10.2%), 57 CNVs (57/579, 9.8%) were classified as variants of uncertain significance (VOUS), and 17 CNVs (17/579, 2.9%) were classified as likely benign (LB) (Table 2).

### 3.1. Anomaly on Ultrasonography

In fetuses with multiple ultrasound defects, detection rates of aneuplidy and triploidy was 21.8% (489/2245) and CNV 9.4% (219/2245). In the subgroup with a structural anomaly in one system, detection rates of aneuploidy and triploidy was 13.7% (269/1959) and CNV 9% (176/1959). The highest percentage of abnormal results was in hydrops fetalis (59/123, 48%), followed by congenital heart disease (285/1188; 24%), or by fetal hypotrophy (19/79, 24.1%) (Table 2).

### 3.2. Advanced Maternal Age (AMA)

A microarray CGH analysis was performed for a total of 336 pregnant women with AMA (over 35 years old), and the average age of this group was 37.65 years (range: 35–49 years). Chromosome abnormalities were detected in 70 samples (70/336, 20.8%), including 46 cases with aneuploidy (46/336, 13.7%), 6 cases with chromosomal triploidy (6/336, 1.8%), and 18 cases with CNV (18/336, 5.4%) (Table 3). Aneuploidies included: 1 case with trisomy 13, 1 case with mosaic trisomy 14, 1 case with trisomy 15, 6 cases with trisomy 18, 35 cases with trisomy 21, 2 cases with monosomy X.

### 3.3. Abnormal Serum Screening Results: PAPP-A

Serum screening results: PAPP-A (first-trimester test), was conducted by measuring pregnancy-associated plasma protein A concentration and the free β-hCG (human chronic gonadotropin) along with the measurement of NT in the USG.

A total of 713 samples with abnormal result in the PAPP-A test were analyzed by microarray CGH, and abnormal results were observed in 136 samples (136/713, 19%), including 110 cases with aneuploidy (110/713, 15.4%) and 26 cases with CNV (26/713, 3.6%) (Table 4).

The 713 samples with abnormal results in the PAPP-A test were divided into four subgroups according to risk type: 615 cases (615/713, 86.3%) with high risk of Down syndrome, 36 cases (36/713, 5%) with high risk of Edwards syndrome, 20 cases (20/715, 2.8%) with high risk of Patau syndrome and 42 cases (42/713, 5.9%) with high risk of trisomy 21, 18 and 13. Among these, microarray confirmed 78 cases in the group of high risk of trisomy 21 syndrome, 13 cases in the group of high risk of trisomy 18, and 5 cases in the group of high risk of trisomy 13. Additionally, in the group of high risk of trisomy 21, we identified 6 cases with chromosome X monosomy. In the group of high risk of trisomy 21, 18 and 13, CGH showed 3 cases with trisomy 21, 3 cases with trisomy 18, and 2 cases with trisomy 13 (Table 4).

### 3.4. Other Indications

In the subgroup with thickened nuchal translucency (NT), the detection rate of abnormal results was 36.9% (212/573). In the subgroup with parental anxiety, we got only correct results in all cases (13/13, 100%) (Table 2).

The last 1533 samples had indications for prenatal testing, such as genetic disorders in family history and verification of the others prenatal tests. The microarray analysis revealed that 60 cases with genetic disorders with family history (60/197, 30.5%) and 418 cases with verification of the others prenatal tests (418/1336, 31.3%) (Table 2) had chromosomal abnormalities.

In 207 cases, indications were based on the family history: mothers who had previous pregnancies with fetal abnormalities or a child with genetic disorders, structural malformations, chromosomal abnormalities or parents who were carriers of a chromosome aberration were also assessed by CMA. In this group, clinically significant CNV was detected in 25 fetuses and numerical aberrations were found in 31 fetuses.

### 3.5. Discrepancy between Karyotyping and CGH Array

Cell culture performed to exclude aberrations not detectable by aCGH method analysis was made simultaneously on 5164 samples (5164/7400, 69.8%) of our cohort. Among 5164 cases with normal aCGH results, routine karyotype analysis showed 31 abnormal results with chromosomal triploidy in the female fetus (31/5264, 0.6%). Chromosomal triploidy was associated with ultrasound disorders: lymphoid holoprosencephaly, collapse of the frontal bones, VSD, abnormal departure of large vessels, omphalocele with displacement, hypotrophy, and feet and hands defects. Additionally, in 34 cases with normal CGH results, karyotype analysis showed balanced translocation or inversion.

Furthermore, CGH analysis showed the presence of microduplication/microdeletion in 265 out of 3474 cases with normal karyotype results.

We identified 57 variants of unknown clinical significance (VOUS). This group covers genetic aberrations are not generally seen in the population and, therefore, have few or no clinical evidence available to evaluate their pathogenic character. The VOUS may include benign familial variants that make no clinical function, or may be rare harmful changes that appear subsequent in a clinical phenotype.

## 4. Discussion

In our study, we focused on the assessment of the usefulness of the aCGH in prenatal diagnosis in the cohort of 7400 pregnancies in a single diagnostic center in Poland. Our research included a large study group mostly thanks to public funds, and strict uniform criteria for qualifying patients for invasive tests. Samples of DNA from both parents were also available in many cases, which significantly helped to classify many CNVs that were difficult to interpret. Genetic counselling was performed before sampling and after obtaining the test results.

The percentage of identified abnormalities was 27.2% (2010/7400), of which 71.2% (1431/2010) were numerical chromosomal aberrations and 28.8% (579/2010) structural aberrations (Table 2). Unfortunately, due to the poor quality of the DNA, it was not possible to obtain reliable results in 126 cases. In our study, the diagnostic success rate was 98.29% (7274/7400). For example, Wang Y. et al., using BACs-on-Beads (BoBs) assay, of the 1520 prenatal cases collected, 1428 cases were successfully analyzed, and 92 cases did not have informative results. The observation rate was 93.95% (1428/1520) [18].

The microarray method not only provides information about the presence of a CNV, but also enables the identification of the chromosomal mosaic. In our study, we identified 28 mosaic chromosomal trisomies and 19 mosaics of X monosomy. Mosaic trisomies of chromosome 8 were identified in 3 cases, chromosome 14 in 3 cases, chromosome 15 in 2 cases, chromosome 16 in 4 cases, chromosome 18 in 3 cases and chromosome 21 in 3 cases. In addition to recurring aneuploidy, we also found a mosaic of chromosomal trisomy in individual cases of chromosomes: 2, 3, 4, 5, 7, 9, 12, 13, 17 and 22. The clinical consequences of chromosomal mosaicism identified during invasive prenatal diagnosis may be difficult to assess, ranging from no apparent clinical phenotype to early fetal death. The chromosomal mosaic detection values are a log2 ratio from 0 to +0.3 or from 0 to −0.5. An example of the lowest mosaic trisomy found in prenatal diagnosis is the 6% mosaic trisomy of chromosome 4 (in 6 out of 100 analyzed cells) confirmed by FISH (from uncultured material). However, during the CGH analysis of this case, the degree of mosaicism was estimated at 25–30% (mean log ration 0.1174). This difference may result from the test procedure itself (for aCGH we used whole genomic DNA, while during the FISH procedure, the entire obtained cell sediment was not used to prepare the preparation, or some cells may not show an informative signal for the probe). Cell culture in a conventional karyotype can promote in vivo selection of euploid cells versus aneuploid cells, which increases with longer culture duration.

Clinically significant structural aberrations (pathogenic and likely pathogenic) accounted for 87.2% (505/579) of all diagnosed CNV and 6.8% (505/7400) of all performed tests. Our detection rate was higher than those presented in other works, for example in the study of Breman A. et al., in which the percentage of clinically significant CNV was 2.4%, or the publication of Chai H. and co-workers, where the detection rate was described as 2.59% [19,20]. However, the results were similar in the group of over 5000 pregnancies described by Shaffer L.G. et al., where the percentage of detected clinically significant copy number changes was 6.5%, in cases referred with abnormal ultrasound examination, while the percentage of results of uncertain clinical significance was 4.2% [21]. In another group of 4282 fetal samples reported by Wapner R.J. et al., the diagnostic efficiency was 6.0% in samples with normal conventional karyotype results, when the indication was a fetal structural defect found on ultrasound and 1.7% when the indication was advanced maternal age or abnormal screening results [15]. Breman A. et al., demonstrated a diagnostic efficiency of 4.2% in a group of 1075 prenatal samples with no known chromosomal abnormalities or familial genomic imbalance [19].

In the study by Lovrecic L. et al., the detection rate of pathogenic CNV in the group of fetuses with defects in ultrasound was 10.0%. The diagnostic efficiency was the highest in the group of cases with multiple congenital malformations (16.7%), and the lowest in the group of isolated IUGR (intrauterine growth restriction) (6.3%) [22]. The study by de Witt M.C. et al., showed that a significant submicroscopic CNV could be identified in 3.1–7.9% of fetuses with a defect limited to one system and in 9.1% of fetuses with multiple abnormalities [23].

**Figure 2:**
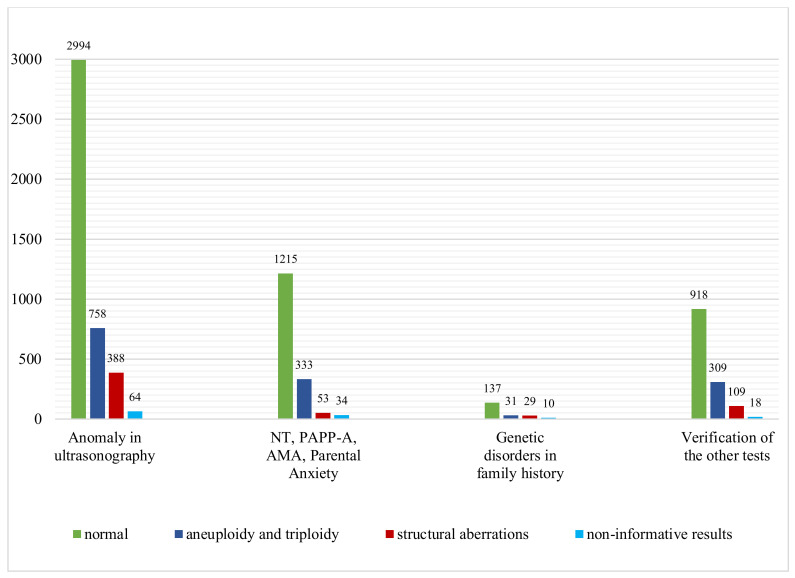
Microarray CGH results of 7400 samples: number of cases with normal results, aneuploidy and triploidy and structural aberrations in four subgroups of different indications for prenatal testing.

All samples received for our study were classified into four groups, according to the indications for invasive prenatal diagnosis: abnormal ultrasound examination result, advanced maternal age, abnormal results of screening test (pregnancy-associated plasma protein A) and parental anxiety, genetic diseases present in the family and the verification of other genetic tests. As expected, the rate of chromosomal abnormalities detected by the CGH microarray was the highest in the group with abnormalities in the ultrasound examination and amounted to 27.3%, and then in the group with advanced maternal age, elevated NT, abnormal PAPP-A test result and anxiety, it amounted to 23.6% (Figure 2).

Microdeletions were found significantly more often than microduplications: 375 and 204 respectively. In our study, clinically significant CNV (pathogenic and likely pathogenic) was detected in 7.6% (149/1959) of fetuses with isolated abnormalities or 8.2% (185/2245) in fetuses with multiple malformations identified during ultrasound examination. Among isolated defects, the highest percentage of CNV was observed in congenital heart defects—10.4%, followed by central nervous system defects with 9.1% or in fetal hypotrophy—8.4%. Studies in other centers have shown that the diagnostic efficiency of CMA in cases of multiple structural abnormalities in the fetus and isolated heart defects ranged from 3.1–10% for multiple abnormalities, and from 7.9–13.1% for isolated defects [21,24,25]. The differences in the degree of detection may be related to the appropriate selection of patients, bias, availability of reimbursed tests or the use of the previously classic karyotype method to exclude microscopically visible chromosomal aberrations in the fetus.

In the group of malformations of one system, the CGH detection rate of all aberration was the lowest for a urinary tract defect and amounted to 4.5% (6/134) including 2.2% (3/134) CNV. In the available literature, the most common pathogenic CNV in fetuses with congenital kidney and urinary tract defects is the 17q12 microdeletion [24,26,27], which causes polycystic kidney disease and the diabetic syndrome. In the study group, three fetuses had urinary tract defects and had a 17q12 microdeletion. In the case of fetal gastroschisis and achondroplasia, we did not find any abnormal results. The genetic causes of these anomalies are heterogeneous and poorly understood. Some of these abnormalities may be due to multifactorial inheritance or a mutation in the single gene, and therefore the use of CGH for diagnosis may be an inappropriate strategy.

**Figure 3:**
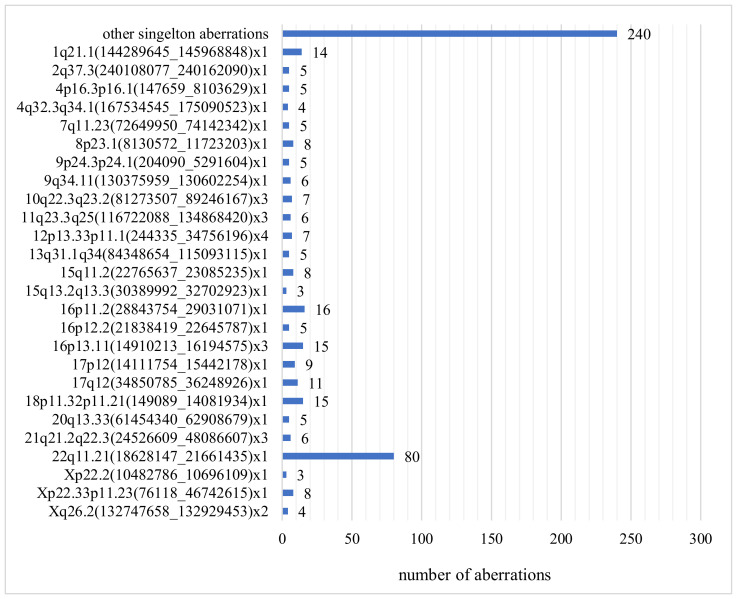
Number and type of clinically relevant CNVs.

The most common submicroscopic aberration in our study was the deletion of the 22q11.21 region (80/7400, 1.1%) (Figure 3). Ultrasound abnormalities in these fetuses included isolated and coexisting heart defects, cleft lip and palate, and congenital anomalies. The second most frequently observed structural aberration was the microdeletion in the 16p11.2 region (16/7400, 0.22%). Abnormalities occurring in these fetuses were increased neck translucency, heart defect, megacystis, ventriculomegaly, cleft lip and cleft palate, and fetal hypotrophy. Postnatal phenotype of 16p11.2 carriers is characterized by motor and speech disorder, language disorder, motor coordination difficulties, psychiatric conditions, and autistic features. If the 16p11.2 deletion is found in the fetus, it is difficult to determine the exact phenotype at birth, most people with a 16p11.2 deletion have language retardation and cognitive impairment. Aberrations with low penetrance are only reported if the USG shows fetal defects corresponding to this deletion.

Duplication of 16p13.11 occurred in 15 fetuses (15/7400, 0.21%) with, inter alia, abnormal results of biochemical test PAPP-A and polysectomy including fetal edema, ascites, and hydrocephalus (Figure 3). The 16p13.11 microduplication syndrome is associated with variable clinical features including behavioral abnormalities, pervasive developmental delay, congenital heart disease, and skeletal defects. As with the 16p11.2 deletion, the prenatally established 16p13.11 microduplication is difficult to interpret and determine the child’s phenotype at birth. Due to low penetrance, 16p13.11 duplication should not be taken into account in the prenatal result, and we did not report it in our results.

The 18p11.32p11.21 deletion was found in 15 fetuses (15/7400, 0.21%) with increased nuchal translucency, heart defect, polydactyly of the hand and multiple lethal malformations: holoprosencephaly, large cleft upper lip, no typical nose, small eyeballs, severe hypertelorism, low nape of the neck, massive edema of the head and thorax (Figure 3). The available literature describes cases of fetuses with increased NT and craniofacial defects.

The 1q21.1 deletion occurred in fourteen fetuses (14/7400, 0.19%), which always had ultrasound findings: hydrocephalus, aqueductal stenosis, pyelectasias, congenital heart defects, skeletal defects, and increased NT (Figure 3). Postnatal carriers of 1q21.1 deletion may present phenotypic variability, e.g., mental retardation, microcephaly, heart defects, short stature, hypotonia, cataract [28,29].

Pathogenic structural CNVs were not detected in the cohort of 573 fetuses with isolated increased nuchal translucency, and in the cohort of 13 fetuses in which the indication was only parental anxiety. Egloff M. et al., described a large group of fetuses with isolated increased neck translucency. In this study, pathogenic CNV was found in only 2.7% of fetuses with a neck translucency ≥3.5 mm, and nearly half of these were CNV associated with neurodevelopmental disorders: autism spectrum disorders, schizophrenia, attention deficit with hyperactivity disorder and intellectual disability [30].

In the group of referrals due to advanced maternal age, clinically significant submicroscopic CNVs were detected in 5.3% (18/336) of fetuses (Table 3), which is higher than the results obtained by Wapner R.J. et al., who found CNV in 1.7% of fetuses tested for AMA [15]. The results of our research showed that the frequency of numerical aberrations increased with age (Table 3), which is in line with previous studies [2]. However, the prevalence of structural aberrations in our cohort with AMA was the highest in the group of patients aged 35–39.

In the group of abnormal serum screening results (PAPP-A), we identified clinically significant submicroscopic CNV in 3.1% (22/713) of the fetuses (Table 4 and Table 5). Our results were higher than results showed by Xiang J et al., in which 2.5% (30/1199) of fetuses had clinically significant CNV [2]. In this group, we identified 22 unexpected, pathogenic structural aberrations. One of the more interesting cases was a female fetus (number 7408), with high risk of trisomy 21 where we identified deletion 5p14.3p14.1, of approximately 5.72 Mb in size. The aberration includes four gene encoding proteins: *CDH18* (OMIM 603019), *CDH12* (OMIM 600562), *PRDM9* (OMIM 6097601 and *CDH10* (OMIM 604555). Deletion of this region has been described in a fetus with congenital heart disease and was inherited from a mother without clinical symptoms [31].

In male fetus (number 7121), with high risk of trisomy 21, we identified a duplication of the 20p12.3p11.1 region (approximately size 20.09 Mb). The test result was confirmed by the FISH method. Analysis of the interphase nuclei by FISH using the RP11-430K20 probe specific for the 20p12.2p12.3 region revealed the presence of two cell lines. In the first line (41.4%), three signals were found for the studied region, and in the second line (58.6%), two signals were found. The duplication included 93 protein-coding genes and was located in the region of the 20p trisomy syndrome (ORPHA: 261318), described in patients with: intellectual disability, speech development delay, impaired motor coordination and facial dysmorphic features. The literature describes a patient with excessive growth, psychomotor retardation, hypotension, features of facial dysmorphia and convulsions, who was diagnosed with mosaic trisomy 20p11.2-p12.1 [32].

In addition to aneuploidies and known recurrent pathogenic CNVs explaining the clinical abnormalities in the fetus, we highlighted 20 interesting structural aberrations that are pathogenic (*n* = 16) (Table 6) or likely pathogenic (*n* = 4) (Table 7) and were associated with the fetus phenotype.

In the group of interesting pathogenic aberrations, we listed two structural aberrations in female fetuses with defects of the central nervous system (case 5653); duplication of 17q12 (approximate size 1.76 Mb) and duplication of Xq28 (approximate size 607 kb). The duplication in 17q12 includes 21 protein coding genes therein: *CCL3L1* (OMIM 601395), *ZNHIT3* (OMIM 604500), *PIGW* (OMIM 610275) and dose sensitive genes: *LHX1* (OMIM 601999), *AATF* (OMIM 608463), *ACACA* (OMIM 200350), *TADA2A* (OMIM 602276), *HNF1B* (OMIM 189907). Additionally, this aberration covers a region of known 17q12 duplication syndrome (OMIM 614526) described in patients among others with: developmental delay, structural brain abnormalities, heart, and kidney defects [33,34]. Duplication of this region found in the fetus with ventriculomegaly and corpus callosum agenesis has been described in the literature, as well as in a patient with Th9 vertebrae of the thoracic spine [34]. Aberration in this region was characterized by variable expression and incomplete penetrance (21.1%) and occurred de novo. Duplication in Xq28 includes 13 protein coding genes for example: *GAB3* (OMIM 300482), dose-sensitive gene *DKC1* (OMIM 300126), *MPP1* (OMIM 305360), *SMIM96*, *F8* (OMIM 300841), *H2AFB1* (OMIM 301037), *F8A1* (OMIM 305423), *FUNDC2* (OMIM 301042), *CMC46*, *MTCP1* (OMIM 300116), *BRCC3* (OMIM 300617), *VBP1* (OMIM 300133) and exon 2 of the *RAB39B* gene (OMIM 300774) and partially encompasses the distal Xq28 microduplication syndrome region (ORPHA: 293939) with candidate genes *RAB39B1* and *CLIC2* (OMIM 300138). Our fetus was the only carrier of the aberration. This syndrome is reported in male patients with intellectual disability, behavioral disorders, facial dysmorphic features, and recurrent infections. Parental aCGH showed that identical aberrations were detected in a normal, asymptomatic father, which was a big surprise for us.

In the male fetus (number 7271), directed to the diagnostics due to advanced maternal age, high risk of chromosomal aberration (high risk of T21 1:29), intrauterine growth restriction, tetralogy of Fallot and syndactyly, we detected deletion 16q24.1 (approximately 653 kb in size). This deletion includes four protein coding genes: dose sensitive *FOXF1* gene (OMIM 601089), *MTHFSD* gene (OMIM 616820), dose sensitive *FOXC2* gene (OMIM 602402), *FOXL1* gene (OMIM 603252) and is located in the region of microdeletion 16q24.1 (ORPHA: 352629). Deletions in the 16q24.1 region involving the *FOX* gene cluster, which were described in patients, involved among others: vascular dysplasia of the pulmonary alveoli with displacement of the pulmonary vessels, heart defects, gastrointestinal malformations and urinary system defects, including hydronephrosis [35,36,37]. The aCGH tests in parents showed that the identified aberration occurred de novo.

The second group of clinically significant aberrations consists of four interesting likely pathogenic CNVs (Table 7). Deletions and duplications were classified as likely pathogenic CNV if they contained candidate genes that may contribute to the abnormal phenotype. The inheritance of these aberrations was established in three cases and they were inherited from a normal mother. One of the examples was a female fetus with the referral reasons: cerebral hydrocephalus, the width of the lateral ventricle 13 mm, concave outline of the frontal bones (symptom of lemon and banana), hernia in the sacro-lumbar section and maternal age 37 (case number 2643). We have identified duplications of 10q24.31q24.32 (approximal size 658 kb) and Xq27.1 (approximal size 565 kb) regions. Duplication of the 10q24.31q24.32 region includes genes: *BTRC* (OMIM 603482), *DPCD* (OMIM 616467), *FBXW4* (OMIM 608071), exons 1–2 of the *TLX1NB* gene (OMIM 612734) and dose sensitive genes: *TLX1* (OMIM 186770), *LBX1* (OMIM 604255), *POLL* (OMIM 606343) and *FGF8* (OMIM 600483). Duplications of this region have been reported in patients with limb defects (SHFM3; OMIM 246560). Duplication in Xq27.1 involves the dose sensitive *SOX3* gene (OMIM 313430). Duplications of this region have been reported in patients with polyhormonal hypopituitarism (OMIM 312000), sex-linked intellectual disability with isolated growth hormone deficiency (OMIM 300123) and neural tube defects [38,39,40]. Parental microarray analysis has shown that the aberrations were inherited from a normal mother.

The aCGH method allows the detection of the entire CNV spectrum, from aneuploidy to very small submicroscopic aberrations, including pathogenic, likely pathogenic, and also those of unknown clinical significance (VOUS). These changes were defined as VOUS in our test group based on the following criteria: CNVs that do not have a clearly defined effect on the clinical phenotype at the time of publication of the test results; includes a gene or genes that have an unknown effect on the identified fetal defect (Table 8). The VOUS were detected in 57 cases, which is 0.7% of all tested fetuses, or 9.8% of fetuses diagnosed with CNV (Table 2). The detection rate of VOUS CNVs in our study was lower than that of a previous multicenter study (1.6%) by Wapner R.J. et al. [15], than that of another cohort of 5026 pregnancies (4.6%) showed in work by Wang J.C. et al., 2019 [41]. These differences could be caused by different aberration reporting criteria and discrepancies in the interpretation of the results.

The VOUS interpretation remains the biggest challenge in the prenatal diagnostics. Therefore, a detailed understanding of clinically relevant genetic variants is very important. Including them in the literature together with the clinical description of the fetal defects can provide the basis for the future classification of variants as pathogenic or benign. Unfortunately, only association studies within the family can determine their exact pathogenic potential. As the potential benefits must be weighed against the possible risks, it is now recommended that only pathogenic or likely pathogenic CNV be disclosed to parents. Showing aberrations with VOUS status in the results can cause a lot of stress and anxiety for parents. Therefore, in all cases where VOUS were detected, according to informed consent, the parents were not informed about the variant and therefore the parental origin of the detected aberration could not be tested.

In conclusion, our studies have shown that aCGH is a very useful method in the diagnosis of fetal defects in a pre-selected high-risk population. Careful and individualized counselling before and after genetic testing is necessary due to the relatively high risk of obtaining outcomes of uncertain clinical relevance, such as copy number changes associated with highly variable expression or incomplete penetrance.

## Figures and Tables

**Table 1:** Number of tests performed in a particular year along with the number of correct, incorrect, and non-informative results.

Year	2014	2015	2016	2017	2018	2019	2020	2021 (Until to the Day 30 September 2021)	Total Results
All Results	71	195	398	733	1191	1710	1812	1290	**7400**
Normal	51	142	276	522	844	1163	1290	976	**5264 ** **(71.6%)**
Abnormal	17	51	110	190	321	539	495	287	**2010 ** **(26.7%)**
Non-informative	3	2	12	21	16	12	33	27	**126 ** **(1.7%)**

**Table 2:** Microarray results of 7400 samples according to reason for prenatal testing.

Indications	Number of Patients	Normal Results	Abnormal Results		Aneuploidy and Triploidy	CNVs	P	LP	VOUS	LB	Non- Informative Results
Achondroplasia	3	3	0	0.0%	0	0	-	-	-	-	0
Gastroschisis	7	6	0	0.0%	0	0	-	-	-	-	1
Parental Anxiety	13	13	0	0.0%	0	0	-	-	-	-	0
Skeletal defects	65	54	10	15.4%	6	4	2	2	-	-	1
Omphalocele	69	52	13	18.8%	8	5	2	1	2	-	4
Fetal hypotrophy	83	60	19	22.9%	12	7	7	-	-	-	4
Hydrops fetalis	123	61	59	48.0%	44	15	15	-	-	-	3
Cranio-facial defects	133	111	22	16.5%	18	4	4	-	-	-	0
Urinary system defects	134	124	6	4.5%	3	3	1	2	-	-	4
Central nervous system	154	121	31	20.1%	16	15	8	3	3	1	2
Genetic disorders in family history	207	137	60	29.0%	31	29	21	4	1	3	10
Advanced Maternal Age	336	256	70	20.8%	52	18	8	10	-	-	10
NT	573	345	212	36.9%	199	13	-	-	4	4	16
Abnormal results of biochemical test PAPP-A	713	601	104	14.6%	82	22	14	8	6	-	8
Congenital heart defects	1188	894	285	24.0%	162	123	94	8	21	-	9
Verification of the others tests	1354	918	418	30.9%	309	109	104	2	-	3	18
Multiple birth defects	2245	1508	701	31.2%	489	212	166	19	20	6	36
**Total results**	**7400**	**5264**	**2010**	**27.2%**	**1431**	**579**	**446**	**59**	**57**	**17**	**126**

(Pathogenic CNVs (P), Likely Pathogenic CNVs (LP), Variants of Unknown Significance (VOUS), Likely benign CNVs (LB), Nuchal translucency (NT)).

**Table 3:** The aCGH results of 336 samples with indication of advanced maternal age.

Age	Number of Patients	Normal Results	Abnormal Results		Aneuploidy and Triploidy		Pathogenic Structural Abnormality	
35–39	191	153	38	19.8%	25	13.1%	13	6.8%
40–44	119	93	26	21.8%	21	17.6%	5	4.2%
45–49	26	20	6	23.1%	6	23.1%	0	0.0%
**Total results**	**336**	**266**	**70**	**20.8%**	**52**	**15.5%**	**18**	**5.4%**

**Table 4:** The aCGH results of 713 samples with abnormal result in PAPP-A test and normal USG.

	Number of Patients	Normal Results	Abnormal Results	Trisomy of a Specific Chromosome		Monosomy X		Structural Aberrations	
High risk of T21	615	514	101	78	12.7%	6	1.0%	17	2.8%
High risk of T18	36	22	14	13	36.1%	-	-	1	2.8%
High risk of T13	20	15	5	5	25.0%	-	-	0	0.0%
High risk of T21, T18, T13	42	30	12	2xT13, 3xT18, 3xT21	19.0%	-	-	4	9.5%
**Total** **results**	**713**	**581**	**132**	**104**	**14.6%**	**6**	**0.8%**	**22**	**3.1%**

**Table 5:** 22 structural aberrations detected in group of 713 samples with abnormal result on PAPP-A test and normal USG.

Patient Number	High Risk of Trisomy	aCGH Results
1362	T21	21q21.1q22.11(19352022_33657166)x3, 21q22.11q22.13(35734654_38189781)x3, 21q22.2(41187502_41429023)x3, 21q22.3(42850375_46139875)x3, 21q22.3(46991188_47561714)x3
1985	T21	Xp22.33p11.23(76118_46742615)x1
2145	T21	22q11.21(18847961_21457610)x3
2219	T21	9p22.3(14747397_15041021)x3
2652	T21, T18, T13	2q22.1q35(142089727_219021345)x3
3356	T21	1p32.3(50817235_52280457)x3 pat
3463	T21	4q32.1q32.2(160399605_163122660)x3 pat
3476	T21	22q11.21(18894820_21457610)x3
3769	T21	Xp11.22(53463247_53790726)x3
4099	T21	1q21.1q21.2(146155929_147824212)x1
5342	T21	22q11.21(21081284_21457610)x1 dn
5672	T21, T18, T13	5p15.33p15.32(22149_4768822)x1, 5p15.2(10212960_12513658)x1, 11p15.5p14.1(113082_27880946)x3
6139	T21	1p13.2p13.1(115761998_116816569)x3
6219	T21	16p12.2(21926361_22407951)x1 mat
7121	T21	20p12.3p11.1(5593060_25678293)x3
7408	T21	5p14.3p14.1(19364195_25086222)x1

(trisomy of chromosome 21 (T21), trisomy of chromosomes 21,18,13 (T21, T18, T13), paternal (pat), maternal (mat), de novo (dn)).

**Table 6:** Examples of interesting pathogenic CNVs which were correlated with the fetal defects.

Case Number	Aberration	Indications for the Study	Size	Sex	Inheritance	Interpretation
1956	17q12(34569737_36290311)x3	cyst in the fetus	1.72 Mb	F	unknown	The aberration covers the region of the known RCAD syndrome (OMIM: 137920), described in patients with MODY diabetes and renal cystic disease. Duplications in the 17q12 region can manifest themselves in a wide spectrum of clinical symptoms.
2652	2q22.1q35(142089727_219021345)x3	screening test showing high risk of chromosomal aberration T 18 1:5, T21 1:5	77 Mb	F	unknown	The aberration spans multiple genes and is responsible for the abnormalities found in the fetus in ultrasound examination.
3426	Xp21.1(32006239_32383121)x0	NT = 3mm	377 kb	M	unknown	The deletion includes exons 36–44 of the *DMD* gene (OMIM 300377). Mutations, deletions of this gene have been reported in patients with Duchenne Muscular Dystrophy (OMIM: 310200) and Becker Muscular Dystrophy (OMIM: 300376).
5027	15q13.2q13.3(30389992_32702923)x1	mitral valve atresia, left ventricular hypoplasia, aortic hypoplasia	2.31 Mb	M	unknown	Deletions of this region have been described in patients mainly with psychomotor retardation, epilepsy, behavioral disorders, less often with discrete dysmorphic features and congenital heart defects (ORPHA: 199318)
5037	16q23.1(75598893_78008020)x1, 17p12(14111754_15442178)x1	hypotrophy, cleft lip	2.41 Mb; 1.33 Mb	F	unknown	The deletion in the 16q23.1 region includes 10 genes encoding proteins: *GABARAPL2* (OMIM 607452), *ADAT1* (OMIM 604230), *KARS* (OMIM 601421), *TERF2IP* (OMIM 605061), *CNTNAP4* (OMIM 610518), *MON1B* (OMIM 608954), SYCE1L2, *ADAMTS18* (OMIM 607512), *NUDT7* (OMIM 609231), *VAT1L2*. The deletion 17p12 region includes 7 genes encoding proteins: *COX10* (OMIM 602125), *CDRT152*, *HS3ST3B1* (OMIM 604058), dose-sensitive *PMP22* gene (OMIM 601097), *TEKT3* (OMIM 612683), *CDRT42*, *TVP23C2* and the critical region of hereditary neuropathy syndrome with hypersensitivity to pressure HNPP (OMIM 162500).
5356	16p12.2(21926361_22645787)x1	screening test showing high risk of chromosomal aberration (T21 1: 18)	720 kb	M	unknown	Additionally, the study revealed a deletion of the short arm of chromosome 16 in the 16p12.2 region of ~720 kb. The aberration covers the region of the known 16p12 deletion syndrome (OMIM 136570), described in patients including with: intellectual disability/psychomotor retardation, congenital heart defects and craniofacial dysmorphic features.
5500	5q35.2q35.3(175116131_180696832)x1	NT = 7.0 mm, heart disease	5.58 Mb	F	unknown	The deletion includes 87 protein-coding genes, including the *NSD1* gene (OMIM 606681) and is located in the region of the 5q35 deletion syndrome (ORPHA: 1627), characterized by: lymphoedema with enlargement of the nuchal translucency in the prenatal period, as well as early childhood hypotension, low height, facial dysmorphic features and heart defects. The 5q35 deletion region includes the region of the Sotos 1 complex (SOTOS1, OMIM 117550), described in patients with: facial dysmorphic features, high birth weight and excess growth early in life, macrocephaly, intellectual disability, as well as heart defects such as atrial septal defect (ASD), ventricular septal defect (VSD).
5653	17q12(34569737_36326421)x3, Xq28(153884022_154491017)x3	defect of the central nervous system	1.76 Mb; 607kb	F	de novo; paternal	Duplication in the 17q12 region includes 21 protein-coding genes, including genes: *CCL3L1* (OMIM 601395), *ZNHIT3* (OMIM 604500) 1, *PIGW* (OMIM 610275) and dose-sensitive genes: *LHX1* (OMIM 601999), *AATF* (OMIM 608463), *ACACA* (OMIM 200350), *TADA2A* (OMIM 602276), *HNF1B* (OMIM 189907) and the duplication syndrome region 17q12 (OMIM 614526). Duplication in the Xq28 region includes 13 protein-coding genes: *GAB3* (OMIM 300482), dose-sensitive *DKC1* (OMIM 300126), *MPP1* (OMIM 305360), *SMIM96*, *F8* (OMIM 300841), *H2AFB1* (OMIM 301037), *F8A1* (OMIM 305423), *FUNDC2* (OMIM 301042), *CMC46*, *MTCP1* (OMIM 300116), *BRCC3* (OMIM 300617), *VBP1* (OMIM 300133) and exon 2 of the *RAB39B* gene (OMIM 300774) and partially the region Xq28 distal microduplication syndrome (ORPHA: 293939)
5843	Xp22.33(76118_1625396)x0 or Yp11.32(27254_1570153)x0, Yq11.221q12(17751498_59329063)x0	screening test showing high risk of chromosomal aberration (T21 = 1:19, T13 = 1:44, T18 = 1:60)	1,55 Mb; 41,58 Mb	M	unknown	The deletion of the Xp22.33 or Yp11.32 region includes 10 protein-coding genes including the *CSF2RA* (OMIM 306250, OMIM: 425000) and *SHOX* (OMIM: 312865, OMIM: 400020) genes. SHOX deletions are pathogenic changes and have been reported in idiopathic short stature patients (OMIM: 300582) (ORPHA: 314795). The identified aberrations indicate the presence of an abnormal Y chromosome.
6219	16p12.2(21926361_22407951)x1	screening test showing high risk of chromosomal aberration (T21 1: 54)	482 kb	F	maternal	The aberration covers the region of the known 16p12 deletion syndrome (OMIM 136570), including 8 genes encoding proteins: *UQCRC2* (OMIM 191329), *PDZD92*, *C16orf522*, *VWA3A2*, *SDR42E22*, *EEF2K* (OMIM 606968), *POLR3E* (OMIM 617815) and *CDR3E* (OMIM 117340). Deletions in the 16p12 region were described in patients with intellectual disability/psychomotor retardation, congenital heart defects and craniofacial dysmorphic features.
6420	15q22.31q26.3(63965478_102383479)x3	brain defect	38.42 Mb	F	unknown	The duplication involves 283 genes encoding the protein, including the 15q overgrowth syndrome (ORPHA: 314585). Partial trisomy 15q has been reported in patients including with facial dysmorphic features, excessive pre- and postnatal growth, kidney defects (e.g., agenesis, hydronephrosis), school difficulties/intellectual disability and behavioral disorders. Additionally, craniosynostosis and macrocephaly are described.
6527	12q24.21(114791285_114846644)x1	AVSD, reduced aortic dimensions	55 kb	M	unknown	The deletion includes the dose sensitive *TBX5* gene (OMIM 601620). Deletions and mutations of this gene have been reported in patients with HOS type 1 syndrome (Holt–Oram syndrome; OMIM 142900) with congenital heart defects and malformations of the upper limbs.
6678	(18)x3,Xq28(154199319_154560374)x3	age 42, cleft palate	trisomy 18; 361 kb	F	unknown	Edwards syndrome. Additionally, the study found duplication in the Xq28 region. The duplication includes 8 genes encoding proteins: exons 1–6 of the *F8* gene (OMIM 300841), *FUNDC2* genes (OMIM 301042), *CMC42*, *MTCP1* (OMIM 300116), *BRCC3* (OMIM 300617), *VBP1* (OMIM 300133), *RAB39B* (OMIM 300774) and exons 2–6 of the *CLIC2* gene (OMIM 300138). The aberration covers the region of distal microduplication syndrome Xq28 (OMIM 300815) (ORPHA: 293939).
7030	17p13.3(736836_1227471)x3	cerebral hernia, VSD, clubfoot	491 kb	F	maternal	The duplication includes exon 1 of the *NXN* gene (OMIM 612895) and the genes *TIMM22* (OMIM 607251), *ABR* (OMIM 600365), *BHLHA9* (OMIM 615416), *TRARG1* (OMIM 612211). Duplications involving the *BHLHA9* gene have been reported in patients with long bone aplasia-associated cleft hand/foot type 3 (SHFLD3) (OMIM 612576) and in patients with a cleft femur-mesomial ectrodactyly (OMIM 228250) (ORPHA: 1986). Less frequently, these patients may develop heart defects, cleft lip and palate, and esophageal atresia2. Duplications of this region are characterized by variable expression and incomplete penetrance (less than 50%).
7271	16q24.1(86053209_86705830)x1	age 35, screening test showing high risk of chromosomal aberration (T21 1:29), multiple defects	653 kb	M	de novo	The deletion includes 4 protein-encoding genes: dose-sensitive *FOXF1* gene (OMIM 601089), *MTHFSD* gene (OMIM 616820), dose-sensitive *FOXC2* gene (OMIM 602402), *FOXL1* gene (OMIM 603252) and is located in the band region 16q24.1 microdeletion (ORPHA: 352629). Deletions in the 16q24.1 region involving the *FOX* gene cluster are described in patients, among others with vascular dysplasia of the alveoli with displacement of pulmonary vessels, heart defects, gastrointestinal defects and urinary tract defects, including hydronephrosis.
7296	16p13.3(3788560_3808214)x1	hypotrophy, NT = 3.5 mm	20 kb	F	unknown	The deletion includes exons 18–26 of the dose-sensitive *CREBBP* gene (OMIM 600140). Deletions and mutations of this gene are described in patients with Rubinstein–Taybi syndrome type 1 (OMIM 180849), who have, among others short stature, postnatal growth retardation, heart defects, skeletal system defects, microcephaly, intellectual disability and dysmorphic features face.

(atrioventricular septal defect (AVSD), nuchal translucency (NT), ventricular septal defect (VSD), female (F), male (M)).

**Table 7:** Examples of interesting likely pathogenic CNVs which can be correlated with the fetal defects.

Case Number	Aberration	Indications for the Study	Size	Sex	Inheritance	Interpretation
1431	11p11.2(43713787_45797075)x3	hypertelorism, asymmetry of the width of the lateral ventricles of the brain, hypoplastic bone of the nose	2.08 Mb	F	maternal	The duplication covers a region of the known 11p11.2 microdeletion (Syndrom Potocki-Shaffer, OMIM 601224).
2558	7p14.1(42058801_42738664)x3	hydrothorax, IUGR	69 kb	F	maternal	The aberration includes exons 1–10 of the dose-sensitive *GLI3* gene (OMIM: 165240), the mutations, deletions and duplications of which have been reported in patients with Greig cephalopolysindactyly syndrome (ang. Greig cephalopolysyndactyly syndrome, GCPS) (OMIM: 175700).
2643	Xq27.1(139283433_139743154)x3, 10q24.31q24.32(102880054_ 103538415)x3	age 37, cerebral hydrocephalus, the width of the lateral ventricle 13 mm, concave outline of the frontal bones (symptom of lemon and banana), hernia in the sacro-lumbar section	658 kb; 565 kb	F	maternal	Duplication in the 10q24.31q24.3 region includes the following genes: *BTRC* (OMIM 603482), *DPCD* (OMIM 616467), *FBXW4* (OMIM 608071), as well as exons 1–2 of the *TLX1NB* gene (OMIM 612734) and dose-sensitive genes: *TLX1* (OMIM 186770), *LBX1* (OMIM 604255), *POLL* (OMIM 606343), and *FGF8* (OMIM 600483). Duplications of this region have been reported in patients with limb defects (SHFM3; OMIM 246560) and may be inherited from the parents. Duplication in the Xq27.1 2 region involves the dose-sensitive *SOX3* gene (OMIM 313430). Duplications of this region have been reported in patients with polyhormonal hypopituitarism (OMIM 312000), sex-linked intellectual disability with isolated growth hormone deficiency (OMIM 300123), and neural tube defects.
3377	16q24.3(89804031_89897059)x1	NT = 4 mm, agenesis of the corpus callosum	93 kb	M	unknown	The deletion includes exons 10–11 of the *ZFP276* gene (OMIM: 608460), exon 1 of the *SPIRE2* gene (OMIM: 609217) and the FANCA gene (OMIM: 607139). *FANCA* gene mutations and deletions have been reported in patients with Group A Fanconi Anemia (OMIM: 227650)

(intrauterine growth restriction (IUGR)).

**Table 8:** Variants of unknown significance and the genes within CNV, which may be responsible for abnormal phenotype.

Case Number	Aberration	Size	Gene/Genes	Indications
48	9q33.2(124229923_124370633)x3	140.71 kb	*OR1J1*	omphalocele, cleft lip and palate
67	7q31.1(110410697_110836614)x1	425.92 kb	*intron 3–4 IMMP2L*, *LRRN3*	NT 7.5 mm, micrognathia, fetal heart defect
193	1q42.2(230763393_231441324)x3, 2q21.1(129829959_131404737)x1	677 kb; 1.57 Mb	*PCNXL2*, *ARHGEF4*	generalized fetal swelling, NT = 3.8 mm, no NB, flat face profile
264	Xp11.4(38096994_38138665)x3	41 kb	*OTC*	family history
303	6q14.3q15(86892972_87919594)x3	1.03 Mb	*HTR1E*	NT = 4 mm, cleft lip
395	5q35.3(177068821_178058571)x3	989 kb	*PROP1*, *NHP2*	hypotrophy, VSD
584	13q13.3(37145323_37351415)x3	206 kb	*SERTM1*	cardiac ectopy
601	7q35(146544277_146840480)x1	296 kb	*ex 4–8 CNTNAP2*	cleft lip, hypotrophy, ASD
602	7q35(146544277_146840480)x1	296 kb	*ex 4–8 CNTNAP2*	widening of the lateral ventricles of the brain, VSD
608	4p15.32(16064173_16813206)x3	750 kb	*TAPT1*, *PROM1*, *LDB*	AVSD
609	4p15.32(16064173_16813206)x3	750 kb	*TAPT1*, *PROM1*, *LDB*	AVSD
674	2p16.3(48059806_48500445)x3	440 kb	*ex 1–10 FBXO11*	tricuspid valve regurgitation
674	2p16.3(48059806_48500445)x3	440 kb	*FBXO11*	tricuspid valve regurgitation
730	8p23.1(11607828_11723203)x3	115 kb	*GATA4*, *NEIL2*, *FDFT1*, *CTSB*	NT
765	11q22.1(101436248_101756583)x3	320 kb	*ex 1 TRPC6*	atrioventricular septal defect (AVSD)
845	2q23.1(149008939_149099960)x1	90 kb	*ex 5 MBD5*	AVSD, duodenal obstruction
851	18q11.1(18542080-18672140)x1	130 kb	*ROCK1*	omphalocele, NT = 4.2 mm
900	8p22(15570685_16812645)x1	1.24 Mb	*MSR1*, *TUSC3*	cystic hygroma, gastroschisis
913	22q11.21(19338815_19584890)x3	246 kb	*HIRA*, *CCDC45*, *UFD1L*	TOF
924	14q32.11(91122067_91681738)x3	559 kb	*TTC7B*, *RPS6KA5*	omphalocele
963	5p15.33(95276_220479)x3	125 kb	*PLEKHG4B*, *LRRC14B*	TOF and no thymus
1045	21q11.2(15824276_16137741)x3	313 kb	*SAMSN1*	Ebstein Syndrome
1093	13q31.3(92065636_92299097)x3	233 kb	*ex 2 GCP5*	ARSA
1165	9q21.32q21.33(86825588_87161409)x3	335 kb	*SLC28A3*	CAT
1198	1q31.1q31.2(188762960_192117536)x3	3.35 Mb	*FAM5C*	abnormal results of PAPP-A test T21 1:8
1278	1p36.32(2633351_3161118)x3	522 kb	*ex 1–3 PRDM16*	abnormal heart rotation
1280	2q14.2(121549137_121659393)x3	110 kb	*GLI2*	AVSD
1641	16q24.3(89584335_90252496)x3 6	668 kb	*TUBB3*	corpus callosum agensy
1658	Xp22.2(11600766_12080374)x2	480 kb	*MSL3*, *ex 1 ARHGAP6*	CHD
1776	2q11.1q11.2(96766564_97643367)x3	876 kb	*NCAPH*, *SEMA4C*	Dandy-Walker syndrome
1889	8q24.11(118391317_118716415)x3	325 kb	*MED30*	omphalocele
1969	Xq22.3(105159857_105621192)x2	460 kb	*SERPINA7*, *ex 16–29 NRK*	NT 4 mm
2033	2p16.3(50831229_50883635)x1	52 kb	*ex 4–8 NRXN1*	hydrocephalus, pyelectasia.
2089	2q31.1(172529289_172676299)x3	147 kb	*ex 10–18 SLC25A12*	cleft lip, VSD
2093	10q26.12(122509983_122668106)x3	158 kb	*WDR11*	Ebstein Syndrome
2133	18q21.31(54832550_55998895)x3	1.17 Mb	*ST8SIA3*, *ONECUT2*, *FECH*, *NARS*, *ATP8B1*, *ex 1–11 NEDD4L*	agenesis of the corpus callosum, concavity of the frontal bones
2195	Xq28(153324080_153362472)x2	30 kb	*ex 2 MECP2*	CHD
2679	5q22.2(112062907_112440503)x3	377 kb	*APC*, *DCP2*, *MCC*, *REEP5*, *SRP19*	abdominal cyst
2715	16q23.3q24.1(84115545_84899135)x3	784 kb	*COLT1*, *DNAAF1*, *MBTPS1*, *USP10*	VSD, renal pyelectasia, PAPP-A test abnormal: intermediate risk of T21 1:10
2824	16p13.11(16041699_16311080)x3	269 kb	*ABCC1*, *ABCC6*	abdominal tumor in the fetus, suspicion of Central Nervous System bleeding.
2889	7p14.2(35241982_35280550)x1	39 kb	*ex 5–8 TBX20*	hyperechoic gut, choroidal plexus cysts.
2909	9p24.3(224412_381572)x1	157 kb	*ex 2–21 DOCK8*	T21 1:56
3011	7q33(137363460_137560000)x3	196 kb	*ex 1–2 DGKI*	CHD
3267	5q35.1(172105222_172352411)x1	247 kb	*ex 1–6 ERGIC1*, *ex 3–5 NEURL1B*, *DUSP1*	hydrocephalus
3356	1p32.3(50817235_52280457)x3	1.46 Mb	*FAF1*, *CDKN2C*, *EPS15*, *OSBPL9*, *ex 14–33 NRD1*	IUGR, VSD
3403	4p15.31(20421696_20673992)x1	252 kb	*ex 5–37 SLIT2*	FGR, hypoplastic NB, shortening of the bones of the long limbs
3433	20q13.33(58725829_60112343)x1	1.39 Mb	*ex 1–2 CDH4*,	NT 4.7 mm
3488	9q33.1(119029804_119319068)x3	289 kb	*ex 9–22 PAPPA*, *19–22 ASTN2*	VSD
5605	6p21.1(42824821_43112733)x3	288 kb	*ex 10–13 GLTSCR1L*, *RPL7L1*, *PTCRA*, *CNPY3*, *GNMT*, *PEX6*, *PPP2R5D*, *MEA1*, *KLHDC3*, *RRP36*, *CUL7*, *MRPL2*, *ex 1–15 PTK7*	TOF
5764	3p26.3(1539221_2757051)x3	1.22 Mb	*ex 1–4 CNTN4*	abnormal results of PAPP-A test T21 1:16
6428	16q23.3(82563542_83763740)x1	1.2 Mb	*ex 1–11 CDH13*	AVSD
6874	6q22.31(123668064_124141121)x3	474 kb	*ex 1 NKAIN2*	abnormal results of PAPP-A test T21 1:45
7055	1p12(120451037_120520297)x1	69 kb	*ex 6–34 NOTCH2*	TOF
7192	11p15.2(14696412_15028562)x1	332 kb	*ex 2–16 PDE3B*, *CYP2R1*, *CALCA*, *CALCB*	NT = 4.0 mm, intestine hyperechoic
7209	15q26.3(100569119_100666644)x1	97 kb	*ex 13–18 ADAMST17*	AVSD, cleft palate
7356	1q43(236761288_236926498)x3	165 kb	*ex 1–4 HEATR1*, ACTN2	age, abnormal results of PAPP-A test T21 1:15
7364	16q23.1q23.2(78658360_79489094)x3	830 kb	*ex 9 WWOX*	abnormal results of PAPP-A test T21 1:43

(Aberrant right subclavian artery (ARSA), common arterial trunk (CAT), congenital heart defects (CHD), nuchal translucency (NT), nasal bone (NB) and tetralogy of Fallot (TOF)).

## Data Availability

Submission of the microarray data to databases may be problematic for ethical reasons, but the data are available upon request to the corresponding author in compliance with EU GDPR.
